# Elbow Reconstruction with Megaprosthesis: An Effective Strategy for Salvage Surgery in Trauma Patients

**DOI:** 10.3390/diagnostics14070724

**Published:** 2024-03-29

**Authors:** Serban Dragosloveanu, Mihnea-Alexandru Petre, Mihai Emanuel Gherghe, Radu Octavian Baz, Romica Cergan, Cristian Scheau

**Affiliations:** 1 Department of Orthopaedics and Traumatology, The “Carol Davila” University of Medicine and Pharmacy, 050474 Bucharest, Romania; 2 Department of Orthopaedics, “Foisor” Clinical Hospital of Orthopaedics, Traumatology and Osteoarticular TB, 021382 Bucharest, Romania; 3 Department of Radiology and Medical Imaging, Faculty of Medicine, “Ovidius” University, 900470 Constanta, Romania; 4 Department of Radiology, “Sf. Apostol Andrei” County Hospital, 900591 Constanta, Romania; 5 Department of Anatomy, The “Carol Davila” University of Medicine and Pharmacy, 050474 Bucharest, Romania; 6 Department of Radiology and Medical Imaging, “Foisor” Clinical Hospital of Orthopaedics, Traumatology and Osteoarticular TB, 021382 Bucharest, Romania; 7 Department of Physiology, The “Carol Davila” University of Medicine and Pharmacy, 050474 Bucharest, Romania

**Keywords:** elbow reconstructive arthroplasty, megaprosthesis, multiple trauma, pseudarthrosis

## Abstract

Delayed fracture healing can have devastating functional consequences, including pseudoarthrosis. Many factors can contribute to delayed healing, including decreased vascularity, micro-motion at the fracture site, large fracture gaps, multiple traumas at the same site, compromised metabolic status, surgical complications, and other conditions. A 61-year-old female patient was referred to our hospital with left distal humeral pseudarthrosis, accompanied by chronic pain and disability. Two years prior, the patient suffered a traumatic incident. At another medical facility, the patient underwent open reduction and internal fixation surgery with simultaneous ulnar nerve transposition. She showed favorable postoperative recovery. Unfortunately, approximately one year later, the patient sustained a second trauma to the same arm. This led to peri-implant fracture and post-traumatic aseptic degradation of the osteosynthesis system which was subsequently removed. Twelve months after the last surgery, the patient was referred to our hospital and, after thorough consideration of the therapeutic options, we decided to perform left elbow arthroplasty with left distal humeral reconstruction by using Zimmer’s Comprehensive Segmental Revision System. This approach is generally reserved for tumors, and only a handful of cases of megaprostheses for non-tumoral indications have been previously reported. The surgery and perioperative care of our patient were optimal, there were no complications, and the patient recovered arm functionality following rehabilitation.

**Figure 1:**
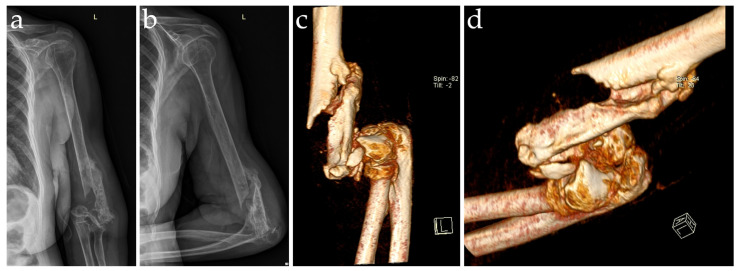
Preoperative anteroposterior (**a**) and lateral (**b**) radiographs and volume rendering technique CT images (**c**,**d**) of a 61-year-old female patient, showing left humeral pseudarthrosis. The patient was referred to our hospital due to a history of left distal humeral multiple traumas, resulting in left distal comminuted, bicolumnar, intra-articular fracture with displacement AO C2/C3 classification, Jupiter Y-type classification and left olecranon Mayo type II displaced non-comminuted stable fracture, AO type B intra-articular fracture, and Schatker simple transverse fracture type A. She underwent multiple open reduction and internal fixation surgeries with ulnar transposition, with medial and lateral plates with screws for the distal humerus fracture and a tension band technique with K-wires for the olecranon fracture, at another hospital. Subsequently, she experienced another traumatic event on the same arm after falling from a height. As a consequence, a peri-implant fracture occurred, alongside the failure of the internal fixation device, leading to the removal of the osteosynthesis materials. The patient exhibited left arm pseudarthrosis, impairing normal function of the elbow joint with persistent pain at the site of the fracture. The patient was referred to our hospital one year after the last surgical procedure. Clinical tests did not highlight any ulnar nerve injury, with no sensory dorsal or palmar dysfunction, no ulnar claw deformity of the hand, and normal motor function. The images show normal proximal radio-ulnar morphology. A CT examination with three-dimensional reconstructions was performed in order to assess the damage to the left distal bone stock and the state of deep tissue fibrosis, as well as to approximately calculate the length of the missing bone segment and the size of the distal humeral reconstruction segment. Both CT exams and X-rays showed severe elbow arthritis, with significant joint space narrowing, osteophytes found at the olecranon tip, and loose bodies. After thorough consideration and analysis in a multidisciplinary board and ruling out subclinical infections by using biological markers and tissue biopsy with joint aspiration, the recommended therapeutic solution was a megaprosthesis, which is usually reserved for tumoral cases but was considered the best approach for this patient [1,2]. The literature on the use of megaprostheses for non-neoplastic indications is scarce, and this novel approach has only been previously described in a handful of very recent cases [3,4]. Poor bone quality and multiple surgeries without clinical neurological deficiency were the main indications for the selected implant. Osteosynthesis, bone reduction, bone grafting, and fixation were not considered as solutions because of previous failures and the possibility of large avascular distal fragments of the humeral shaft that made bone integration unlikely.

**Figure 2:**
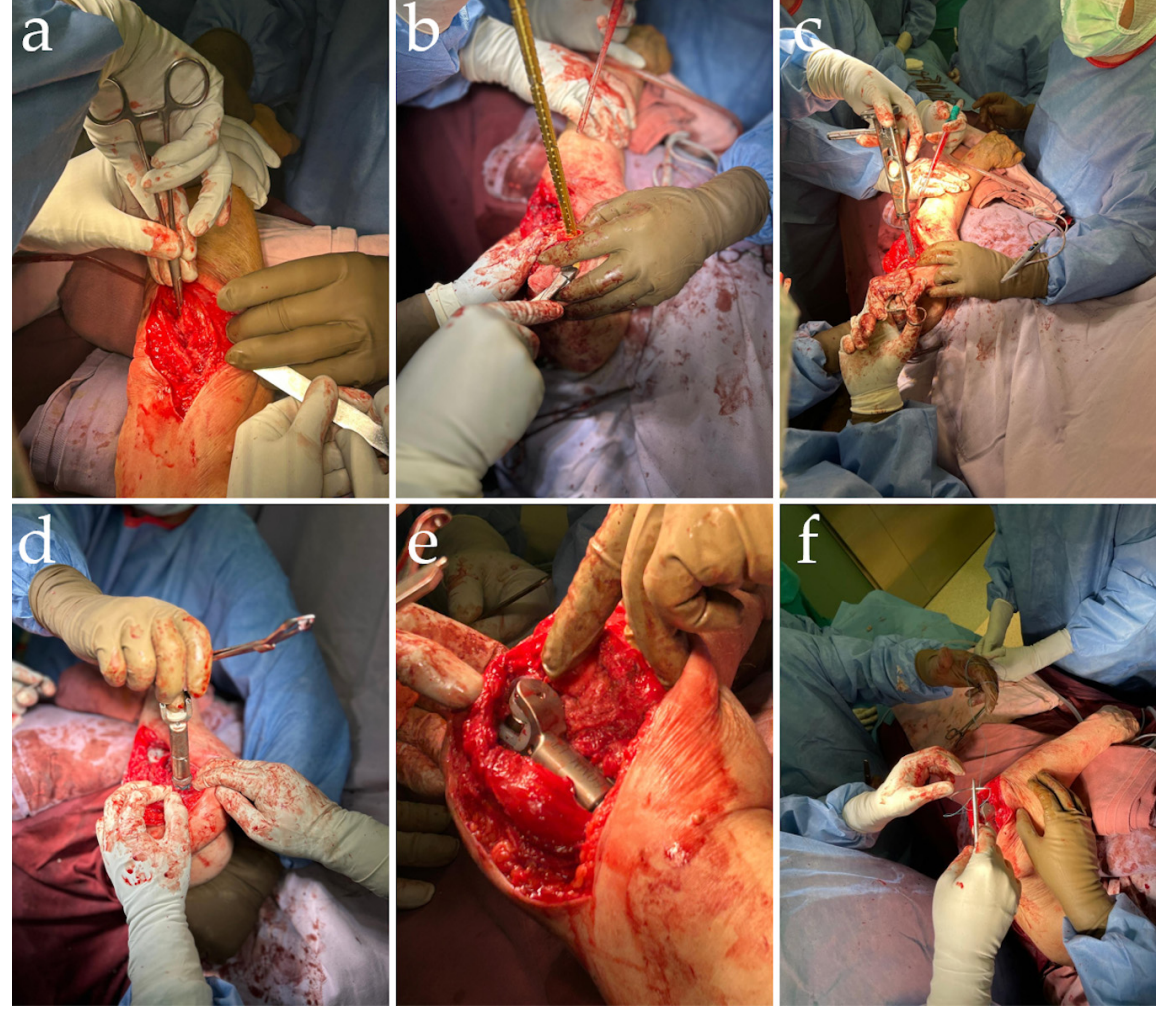
Intraoperative views. The patient was indicated for surgery following the diagnosis of pseudoarthrosis and considering all therapeutic options [5,6,7,8]. The patient underwent general anesthesia, in the supine position, with her left arm crossing her body. The skin was prepared by using regular pre-surgical procedures and subsequently draped. A posterior approach with a triceps-splitting anconeus was chosen for this case because it allows for direct identification of the radial nerve and sufficient exposure of the distal humeral shaft. This approach was performed on the previous incision without osteotomy of the olecranon due to the extent of fibrosis and in order to mitigate the risk of intersecting the ulnar nerve. Following skin incision, careful dissection of the soft tissue and excision of the fibrotic tissue and bone segment were performed (**a**). The next step was preparing the distal humeral resection site, measuring 3 cm from the tip of the distal humeral bone, with progressive drills (**b**) and rasps (**c**). After this step, ulnar preparation was ensured with progressive drills and rasps. After proper fitting components were chosen for the distal humerus and proximal ulna, we assessed the range of motion of the modular prosthesis (**d**,**e**). The trial components were removed, and the final cemented components were installed, using cement restrictors for both components (the distal humerus and the ulnar implants). We utilized anatomical landmarks to approximate the proper rotation of the implant. Considering both variations in the shape of the humeral diaphysis and the section of the humeral implant stem, we considered that a relatively low anteversion could result in a degree of better stabilization, as there is a considerable risk that forcing anteversion or retroversion will fracture the humeral shaft when impacting the stem. We followed literature data that propose that a minor degree of anteversion is generally well tolerated [9]. We used an implant segment of 3 cm to restore anatomical humerus length, and we attached the fibrotic tissue to the anterior flange by using non-absorbable sutures. A bone graft under the flange was not used due to there being no contact between the graft and proximal humerus; also, there were concerns regarding large bone tissue that may be associated with septic complications. The deep layers of the triceps muscle were sutured with non-absorbable suture and then the deep layer of the skin and the skin, in a multilayered fashion (**f**).

**Figure 3:**
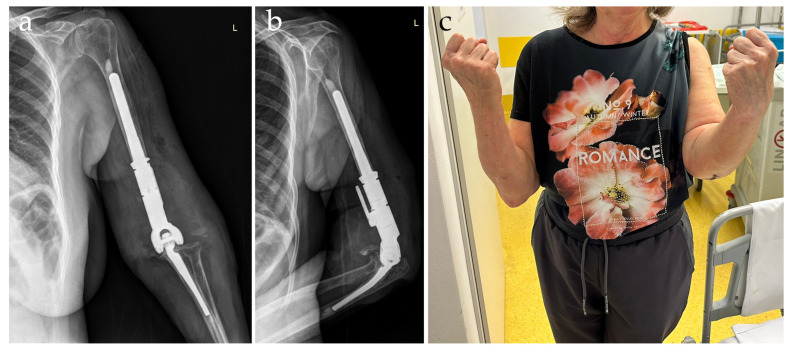
Postoperative radiographic images taken the next day after surgery in anteroposterior (**a**) and lateral (**b**) views. The patient underwent an intensive recovery protocol under permanent analgesia, minimizing the administration of opioids, with a single dose of morphine administered postoperatively. Mobilization exercises were encouraged on the first day after surgery, leading to the recovery of elbow flexion of more than 90° after the first 3 days (**c**). Following surgery, the patient was instructed to start an intensive physical therapy program, to not lift objects heavier than 3 kg, perform proper wound cleaning, and apply ice twice per day for 20 min for the first two weeks. Her recovery was quick, and the patient recovered her functional status by 4 weeks. One month after the total elbow arthroplasty, the patient achieved a flexion range of 110–115 degrees, full extension, and supination of 70–80 degrees.

## Data Availability

The original contributions presented in this study are included in the article; further inquiries can be directed to the corresponding author.
